# Diverse Galactooligosaccharides Differentially Reduce LPS-Induced Inflammation in Macrophages

**DOI:** 10.3390/foods11243973

**Published:** 2022-12-08

**Authors:** Congcong Sun, Bifang Hao, Daorui Pang, Qian Li, Erna Li, Qiong Yang, Yuxiao Zou, Sentai Liao, Fan Liu

**Affiliations:** 1 Sericultural & Agri-Food Research Institute, Guangdong Academy of Agricultural Sciences/Key Laboratory of Functional Foods, Ministry of Agriculture and Rural Affairs/Guangdong Key Laboratory of Agricultural Products Processing, Guangzhou 510600, China; 2 School of Biotechnology, Jiangsu University of Science and Technology, Zhenjiang 212018, China

**Keywords:** galactooligosaccharide, RAW264.7 cells, lipopolysaccharide (LPS), anti-inflammatory activity, TLR4/NF-κB signaling pathway

## Abstract

The effects of natural and synthetic galactooligosaccharides (GOS) on inflammation were explored by investigating the structure-activity relationship between the degree of GOS polymerization and in vitro anti-inflammatory activity, together with the potential underlying mechanism of their anti-inflammatory effects. The results demonstrated that GOS had strong anti-inflammatory effects in lipopolysaccharide (LPS)-induced RAW264.7 macrophages, including the inhibition of nitric oxide production and the reduced expression of pro-inflammatory mediators (interleukin-1β, interleukin-6, and tumor necrosis factor α), induced nitric oxide synthase (iNOS), cyclooxygenase 2 (COX-2), and proteins related to the Toll-like receptor 4 (TLR4)/nuclear factor (NF)-κB signaling pathway. GOS4, which has the highest degree of polymerization, exerted the strongest anti-inflammatory activity among the GOS examined. More importantly, our findings confirmed the anti-inflammatory effects of GOS on RAW264.7 macrophages via the TLR4/NF-κB pathway. Our experimental results could provide further support for the exploration of GOS in human nutrition and health.

## 1. Introduction

Galactooligosaccharides (GOS), a type of functional oligosaccharide with natural properties, are present mainly in the milk of mammals [1]. In addition, GOS are distributed in natural plants, such as the Raffinose family of oligosaccharides, whose common GOS include verbascose, stachyose, and raffinose [2]. GOS have the characteristics of strong acid resistance, heat resistance, good water solubility, and low viscosity; they also display many bioactivities [3].

As typical prebiotics, GOS modify numbers of intestinal bacteria to maintain the microecological balance, thereby enhancing intestinal barrier function [4,5]. GOS could also reduce intestinal permeability and prevent the migration of pathogens [6,7]. In addition, GOS is converted in the intestine into bioactive short-chain fatty acids [8,9], which act as important signaling molecules in regulating blood lipid metabolism via the gut-liver-brain axis [10,11,12,13]. Research also indicates that GOS may exhibit anti-inflammatory activity [14,15]. Indeed, because of their superior physicochemical properties and biological activities, GOS have become a research hotspot in recent years.

GOS are conjugated oligosaccharides consisting of two to eight or more galactose units [16]. A previous study showed that when a GOS mixture was used as the culture medium, B. lactis DR10 preferentially took advantage of GOS with a higher polymerization degree [17]. In addition, GOS with a polymerization degree of three to eight were preferentially utilized by in vitro isolates of infant intestinal flora [18]. These results indicate that GOS with a high polymerization degree can produce higher levels of prebiotic activity. Hence, the anti-inflammatory activity of GOS might be positively correlated with their polymerization degree.

An inflammatory response is a defensive reaction of the body to stimulation. However, if the inflammatory response is not eliminated, inflammation will worsen with the passage of time and may even develop into systemic chronic inflammation, leading to the occurrence of chronic disorders such as autoimmune diseases, cancer, cardiovascular and cerebrovascular diseases, neurodegenerative diseases, and diabetes [19]. For example, when stimulated by lipopolysaccharide (LPS), macrophages secrete pro-inflammatory enzymes such as iNOS and COX-2. Macrophages may also release inflammatory mediators such as IL-1β, TNF-α, nitric oxide (NO), and IL-6. The production of these pro-inflammatory factors is principally adjusted through the NF-κB pathway. Long-term activation of NF-κB signaling downstream effectors may give rise to a vicious cycle of pro-inflammatory signals, leading to persistent or chronic inflammation and, ultimately, chronic diseases. Therefore, it is an effective method to downregulate the expression of these inflammatory factors and proteins in the NF-κB pathway when alleviating inflammatory responses and treating chronic diseases.

An inflammatory cell model was established by inducing RAW264.7 macrophages with LPS in this study. Alteration of inflammatory factors and activation or inhibition of NF-κB inflammatory signaling were observed to elucidate the relationship between the polymerization degree of GOS and their anti-inflammatory activity, as well as potential anti-inflammatory mechanisms. Our findings provide a theoretical basis for further evaluating oligosaccharides in the development of functional food ingredients and tailor-made GOS products.

## 2. Materials and Methods

### 2.1. Materials

Standards of natural GOS (raffinose, stachyose, and verbascose) were purchased from Shanghai Yuanye Bio-Technology (Shanghai, China). The galactose (GOS1) standard was purchased from LGC Labor GmbH (Augsburg, Germany). Galactooligosaccharides standards (Galactobiose-GOS2, Galactotriose-GOS3, and Galactotetraose-GOS4) were obtained from Shanghai Zhenhuai Biotechnology (Shanghai, China). LPS (E. coli, O55:B5) was bought from Sigma-Aldrich (St. Louis, MO, USA). Fetal bovine serum (FBS), double antibiotics (5000 U/mL penicillin and 5000 μg/mL streptomycin), and Dulbecco’s Modified Eagle Medium (DMEM) were bought from Gibco/Life Technologies (Carlsbad, CA, USA). A Cell Counting Kit 8 (CCK-8) was obtained from MedChem Express (Princeton, NJ, USA). Berberine hydrochloride (Ber) was obtained from Shanghai Huangxiang Tieli Lantian Pharmaceutical (Tieli, Heilongjiang, China). Enzyme-linked immunosorbent assay (ELISA) kits for mouse TNF-α, IL-1β, and IL-6 were obtained from Neobioscience Technology (Shenzhen, China). An NO assay kit was bought from Beyotime Institute of Biotechnology (Shanghai, China). GAPDH, iNOS, COX-2, IL-6, IL-1β, and TNF-α primers were bought from Tsingke Biological Technology (Beijing, China). Primary antibodies against β-actin (Cat. No. AC026), Toll-like receptor 4 (TLR4, Cat. No. A5258), IκBα (Cat. No. A1187), and RelA (Cat. No. A10609) were purchased from ABclonal (Wuhan, China). Antibodies against phosphorylated NF-κB (p-NF-κB, Cat. No. 310013) and phosphorylated IκBα (p-IκBα, Cat. No. 340776) were supplied by ZenBioScience (Chengdu, China).

### 2.2. Cell Culture

Mouse monocyte-macrophage RAW 264.7 cells (National Collection of Authenticated Cell Cultures, SCSP-5036) were kindly provided by the Cell Bank of the Chinese Academy of Sciences (Shanghai, China). The cells were cultured in DMEM with 1% double antibiotics (penicillin and streptomycin) and 10% fetal bovine serum at 37 °C and 5% CO_2_. During the logarithmic growth phase, cells were scraped off with a cell spatula and used for experiments.

### 2.3. Cell Viability

RAW264.7 macrophages were seeded in a 96-well plate at a density of 2 × 10^5^ cells/mL (100 μL per well) and divided into blank, control, and GOS (natural GOS and GOS1-4; 18.75, 37.5, 75, 150, 300, and 600 μmol/L) groups. After incubation for 24 h, RAW264.7 cells were treated with 100 μL of vehicle or GOS samples for 24 h. Next, diluted CCK-8 reagent was added into each well and incubated at 37 °C for 30 min. According to the CCK-8 assay instruction, the cell viability was calculated. 

### 2.4. Determination of NO, IL-1β, TNF-α, and IL-6 Levels

RAW264.7 macrophages were seeded in a 24-well plate at a density of 2 × 10^5^ cells/mL (1 mL per well). After 24 h of adherent growth, cells were co-incubated with 300 μmol/L of GOS samples (natural GOS and GOS1-4) and LPS (1 μg/mL) for 24 h, except for control group. Subsequently, cell culture media was collected. According to the NO and ELISA assay kits instructions, the concentrations were quantified respectively.

### 2.5. Isolation of RNA and RT-PCR

After 24 h of treatment, the supernatant was collected and then the cells were harvested after three rinses with phosphate-buffered saline (PBS). Trizol Reagent (Cat No. 15586; Invitrogen, Carlsbad, CA) was used to extract the total RNA from cells. Next, cDNA was generated using a PrimeScriptTM RT Reagent Kit with gRNA Eraser (Cat. No. RR0047A; Takara, Kusatsu, Japan). Real-time PCR was performed with a SYBR FAST qPCR Master Kit (Cat. No. KK4610; Kapa Biosystems, Wilmington, MA, USA). Target genes expression was normalized to GAPDH and was calculated using the 2^−∆∆CT^ method. Primers for qPCR are shown in Table 1.

### 2.6. Western Blotting Analysis

RAW 264.7 macrophages were incubated with GOS as described above, washed three times with PBS, harvested, and lysed with ice-cold radioimmunoprecipitation assay buffer (Beyotime Institute of Biotechnology). The total protein concentration was determined using a bicinchoninic acid protein assay kit, and then was resolved by electrophoresis with 8% and 10% sodium dodecyl sulfate polyacrylamide gels. The gels were transferred to polyvinylidene difluoride membranes. Subsequently, membranes were blocked with 5% skim milk for two hours, followed by the primary antibodies being diluted overnight at 4 °C. Next, membranes were incubated with corresponding secondary IgG antibodies for 1 h. Finally, membranes were incubated with Pierce™ ECL Western (Thermo Fisher Scientific, Waltham, MA, USA), imaged, and analyzed with ImageJ software to determine gray values.

### 2.7. Statistical Analysis

Results were analyzed with SPSS 21.0 (SPSS, Chicago, IL, USA) and expressed as mean ± standard deviation (*n* = 3). ImageJ software (http://imagej.nih.gov, accessed on: 21 July 2021), GraphPad Prism 7 software (GraphPad Software, San Diego, CA, USA), and Biorender (http://biorender.com, accessed on: 9 October 2022) were used to visualize data. The significant differences (*p* < 0.05 or *p* < 0.01) between groups were determined using one-way analysis of variance (ANOVA) combined with Duncan’s test.

## 3. Results

### 3.1. Effects of Natural GOS on Cell Viability 

**Figure 1:**
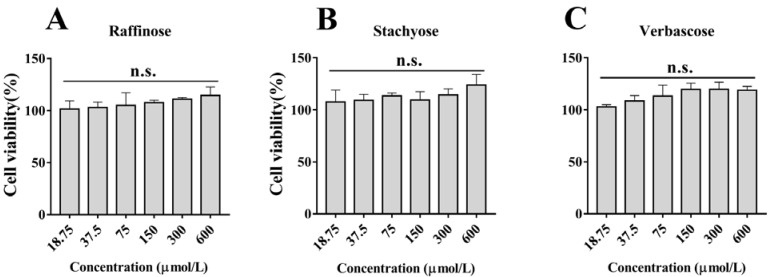
Effects of different concentrations of natural GOS (raffinose, stachyose and verbascose) on cell viability of RAW264.7 macrophages after treatment for 24 h: (**A**) Raffinose, (**B**) Stachyose and (**C**) Verbascose. N.s., not significant (*n* = 3).

Cells were treated with different concentrations of natural GOS (raffinose, stachyose and verbascose) for 24 h. As shown in Figure 1, the results of the CCK-8 assay demonstrated that the cell viability was close to 100% at GOS concentrations under 600 μmol/L, indicating that natural GOS was not cytotoxic to RAW264.7 macrophages below this concentration. According to our experimental results, 300 μmol/L of natural GOS was selected for subsequent experiments, taking activity and economics into account.

### 3.2. Effects of Natural GOS on NO, IL-1β, TNF-α, and IL-6 Secretion

To examine the effect of natural GOS on inflammation, we evaluated levels of inflammatory factors NO, IL-1β, TNF-α, and IL-6 in LPS-induced RAW264.7 cells. As shown in Table 2, after LPS stimulation, expression of inflammatory cytokines was significantly increased (*p* < 0.05, vs. Control). Interestingly, raffinose, stachyose, and verbascose (with polymerization degrees of three to five, respectively) sharply reduced the secretion of inflammatory factors and produced significantly different anti-inflammatory activities in LPS-induced RAW264.7 cells (*p* < 0.05). Among them, the anti-inflammatory activity of verbascose was the strongest, followed by stachyose and raffinose. Therefore, we preliminarily inferred that the degree of GOS polymerization (i.e., number of galactosyls) greatly influenced their anti-inflammatory activities.

### 3.3. Effects of GOS1-4 on NO, IL-1β, TNF-α, and IL-6 levels

**Figure 2:**
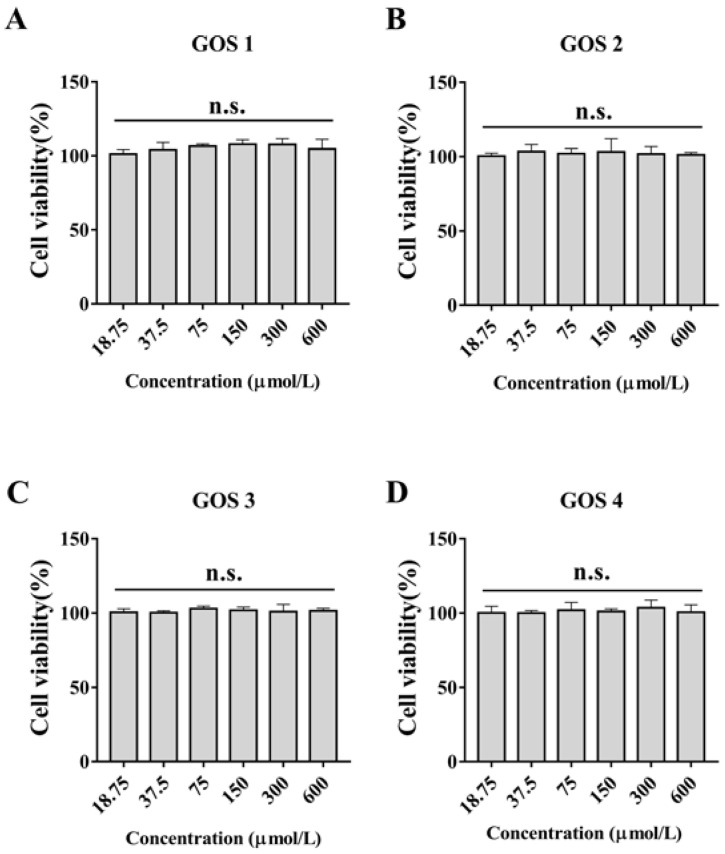
Effects of different concentrations of GOS1-4 on cell viability of RAW264.7 macrophages after treatment for 24 h: (**A**) GOS1, (**B**) GOS2, (**C**) GOS3, and (**D**) GOS4. N.s., not significant (*n* = 3).

To further study the relationship between the polymerization degree and anti-inflammatory activity of GOS, synthetic GOS (GOS1-4) consisting of simple structures (only galactosyl units) were selected. First, we performed a CCK-8 assay to examine the effect of GOS1-4 on cell viability. As shown in Figure 2, treatment of RAW 264.7 cells with GOS1-4 (under 600 μmol/L) did not affect cell viability. Taking activity and economics into account, 300-μmol/L concentrations of GOS1-4 were ultimately selected to further study their anti-inflammatory effect and underlying mechanisms.

**Figure 3:**
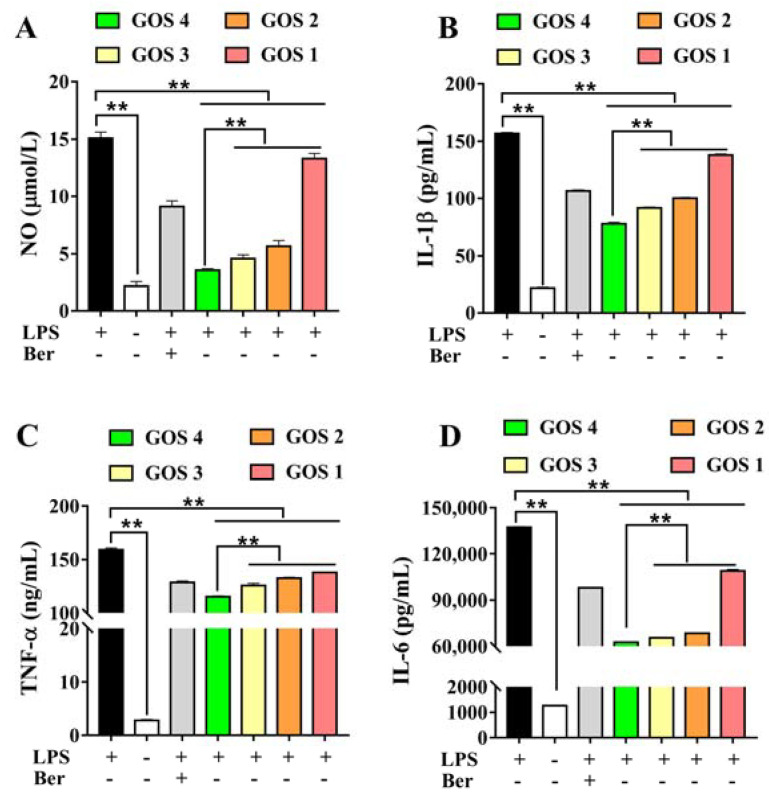
Effects of GOS1-4 on levels of inflammatory mediator in RAW264.7 cells induced by LPS: (**A**) NO, (**B**) IL-1β, (**C**) TNF-α, and (**D**) IL-6. One-way ANOVA combined with Duncan’s test, ** *p* < 0.01, the difference was statistically significant.

In response to external stimuli, the body produces an inflammatory response and increases the release of NO. As shown in Figure 3A, after LPS induction, NO release was significantly increased (*p* < 0.01). After treatment with GOS1-4, NO levels were dramatically decreased (*p* < 0.01). Notably, treatment with GOS4 reduced the level of NO to a greater extent compared to the other treatment groups (*p* < 0.01). We speculated that the decrease of NO was positively correlated with the polymerization degree of GOS. Based on our results, GOS4 with the highest polymerization degree had the strongest inhibitory effect on NO release, consistent with the results of natural GOS.

IL-1β, TNF-α, and IL-6 levels were evaluated to investigate the effect of GOS on the inhibition of inflammatory cytokines. The results showed that LPS significantly induced the secretion of the inflammatory factors IL-1β, TNF-α, and IL-6 (*p* < 0.01). However, after treatment with GOS, levels of inflammatory mediators were significantly decreased (*p* < 0.01). Moreover, levels of IL-1β, TNF-α, and IL-6 were significantly different in GOS1-4 groups. GOS4, which has the highest degree of polymerization, most strongly inhibited the secretion of IL-1β, TNF-α, and IL-6 (*p* < 0.01) (Figure 3B–D), indicating that the degree of GOS polymerization affected their anti-inflammatory activity.

### 3.4. Effects of GOS1-4 on Pro-inflammatory Cytokine mRNA Expression in LPS-Induced RAW264.7 Macrophages

**Figure 4:**
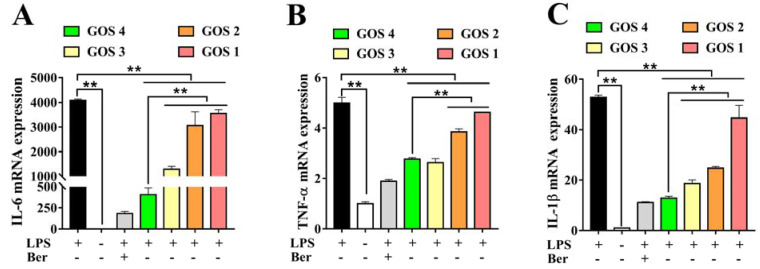
Effects of GOS1-4 pro-inflammatory cytokine mRNA expression in LPS-induced RAW264.7 macrophages: (**A**) IL-6, and (**B**) TNF-α, and (**C**) IL-1β. One-way ANOVA combined with Duncan’s test, ** *p* < 0.01, the difference was statistically significant.

The mRNA expression levels of several pro-inflammatory cytokines, including TNF-α, IL-6, and IL-1β, were detected by quantitative real-time PCR in LPS-stimulated RAW264.7 macrophages. LPS significantly increased the levels of all three pro-inflammatory cytokines in comparison with the control group (*p* < 0.01) (Figure 4). Concurrently we found that LPS stimulation led to a 4000-fold increase in IL-6 mRNA compared to the control group (Figure 4A). Furthermore, GOS with different degrees of polymerization (qualitative anti-inflammatory activity order: GOS4 > GOS3 > GOS2 > GOS1) produced anti-inflammatory activities by reducing pro-inflammatory cytokines IL-6, TNF-α, and IL-1β mRNA expression levels in LPS-stimulated RAW264.7 macrophages compared with the LPS group (Figure 4A–C). GOS elicited potent anti-inflammatory activities by reducing pro-inflammatory cytokines genes expression levels. In brief, GOS4 demonstrated an excellent anti-inflammatory capacity compared with GOS1-3. This result was extremely consistent with the decrease of inflammatory factors induced by GOS with varying degrees of polymerization.

### 3.5. Effects of GOS1-4 on Pro-inflammatory Enzyme mRNA Expression in LPS-Induced RAW264.7 Macrophages

**Figure 5:**
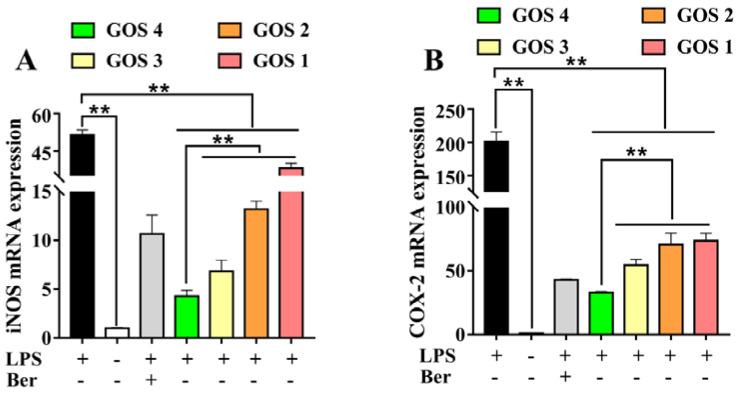
Effects of GOS1-4 on proinflammatory enzyme mRNA expression in LPS-induced RAW264.7 macrophages: (**A**) iNOS, and (**B**) COX-2. One-way ANOVA combined with Duncan’s test, ** *p* < 0.01, the difference was statistically significant.

Following stimulation of cells with LPS, expression levels of pro-inflammatory cytokines increased, leading to the secretion of iNOS and COX-2 [20]. As shown in Figure 5, a dramatic increase in iNOS and COX-2 mRNA was observed following the induction of cells by LPS (*p* < 0.01). After the treatment of the cells with GOS1-4, iNOS and COX-2, mRNA expression levels were decreased to varying degrees (*p* < 0.01). Moreover, these results showed that the inhibition of pro-inflammatory enzyme iNOS and COX-2 mRNA expression by GOS4 was stronger than that elicited by GOS1-3 (*p* < 0.01).

### 3.6. Potential of GOS to Modulate the TLR4/NF-κB Signaling Pathway

**Figure 6:**
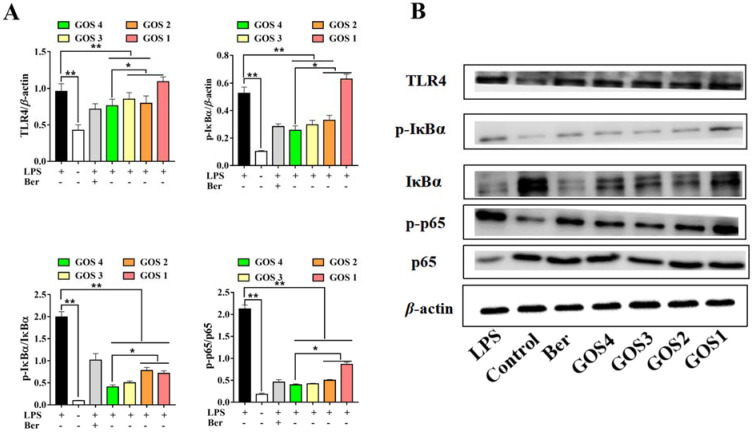
Effects of GOS1-4 on the TLR4/NF-κB signaling pathway in LPS-induced RAW264.7 macrophages: (**A**) The levels of protein expression were assessed by western blot; (**B**) Representative image of western blotting. One-way ANOVA combined with Duncan’s test, * *p* < 0.05, ** *p* < 0.01, the difference was statistically significant.

Macrophage TLR4 levels were observed to be dramatically increased with LPS induction. After GOS pretreatment with different degrees of polymerization, the expression of TLR4 was significantly decreased in RAW264.7 cells (*p* < 0.01), with the exception of GOS1. In particular, GOS4 could significantly reduce TLR4 expression (*p* < 0.05). In addition, the expression and corresponding phosphorylated forms of NF-κB pathway components IκBα and p65 were analyzed by western blotting analysis. IκBα and NF-κB p65 phosphorylation levels were overexpressed in cells following their stimulation by LPS (*p* < 0.01) (Figure 6). However, GOS preincubation significantly decreased IκBα phosphorylation (*p* < 0.01) compared to LPS alone. Moreover, GOS4 demonstrated an excellent capacity for inhibiting IκBα phosphorylation compared with GOS1-3. The results also showed higher p-NF-κB p65 levels in the LPS group (*p* < 0.01), which indicated that NF-κB was active. Similar to our results for proinflammatory cytokines, the higher the polymerization degree of GOS the more significant the inhibition of p-p65 levels. Collectively, our results suggest that GOS4, which had the highest polymerization degree among GOS1-4, could exert the strongest anti-inflammatory effect in suppressing TLR4/NF-κB signaling.

## 4. Discussion

As an important immune cell of the body, macrophages play a crucial part in the body’s defense process. When evoked by external stimuli, such as LPS, macrophages secrete large amounts of inflammation factors and inflammatory mediators [21], thereby causing an inflammatory response. Prolonged duration and abnormal resolution of inflammation have been implicated in the occurrence of chronic diseases [22,23]. Accordingly, it is necessary to treat these diseases by modulating macrophage-mediated inflammatory responses.

As a functional biomolecule, GOS has been shown to reduce oxidation, protect the liver, and regulate neuroinflammation and cognitive impairment [24,25]. In addition, GOS could effectively improve ulcerative colitis induced by sodium dextran sulfate [14,26]. Similarly, our experiments show that natural GOS of the Raffinose family could significantly reduce the secretion of inflammatory factors, suggesting that they have good anti-inflammatory activity. Moreover, our results indicated a qualitative anti-inflammatory activity order: verbascose > stachyose > raffinose (Table 2). We speculated that the degree of GOS polymerization greatly influenced their anti-inflammatory activity. Therefore, a more intensive study of the relationship between the in vitro anti-inflammatory activity and their polymerization, as well as potential anti-inflammatory mechanisms in vitro were explored.

The inflammatory cytokines IL-1β, IL-6, and TNF-α play a vital role in the development of inflammation and correlate with the degree of inflammation [27,28]. Moreover, cells induced by LPS secrete higher levels of pro-inflammatory enzymes, such as COX-2 and iNOS [29,30,31]. iNOS, which is highly expressed in macrophages after exposure to bacterial endotoxins (such as LPS) or pro-inflammatory cytokines, leads to dramatically increased production of NO, which exacerbates inflammation [32]. After LPS stimulation, COX-2 levels were significantly increased; moreover, production of the inflammatory mediator prostaglandin E2 by the enzymatic action of COX-2 further prompted inflammation [33]. Therefore, suppressing the overexpression of pro-inflammatory cytokines and enzymes could effectively alleviate the inflammatory response, thereby producing anti-inflammatory activities.

In this research, GOS were assessed for their anti-inflammatory effects using LPS-induced RAW264.7 macrophages as an inflammatory model. Specifically, we measured the production of pro-inflammatory factors following the incubation of RAW264.7 macrophages with GOS. Using ELISA and NO assays, we found that, compared to the LPS group, GOS obviously reduced the release of inflammatory mediators such as NO, TNF-α, IL-1β, and IL-6 (Figure 3). Moreover, qRT-PCR revealed that GOS could significantly suppress the mRNA expression of IL-6, TNF-α, and IL-1β (Figure 4). Similarly, compared to the LPS group, the mRNA expression of pro-inflammatory enzymes COX-2 and iNOS was dramatically decreased (Figure 5). Taken together, these results further demonstrate that GOS could significantly alleviate inflammatory reaction induced by LPS stimulation, thereby exerting anti-inflammatory activities to inhibit the development of inflammation.

**Figure 7:**
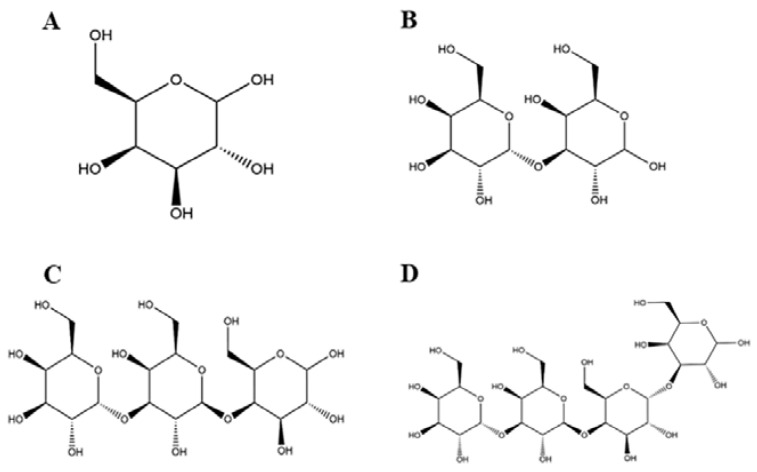
The chemical structure of GOS1-4 (**A**–**D**).

Studies have shown that neoagarotetraose could dramatically decrease the expression of iNOS and pro-inflammatory factors such as IL-6 and TNF-α [34]. Meanwhile, the chitosan oligosaccharide with DP 7 exerted the highest antioxidant and anti-inflammatory activity [35]. More importantly, the in vitro anti-glycation activity of pectin oligosaccharides has positive correlation with the content of oligogalacturonide, and their prebiotic activity shows positive correlation with the content of arabinose and galactose [36]. In this regard, it is speculated that the content of galactosyl units might affect the biological activity of GOS. GOS1-4 are composed of one to four galactosyl groups respectively, as shown in Figure 7. Thus, we speculated that the number of galactosyl units (i.e., degree of polymerization) could affect the anti-inflammatory activity of GOS in vitro. Our results indicated that the releases of NO, IL-1β, IL-6, and TNF-α were significantly increased in RAW264.7 macrophages induced by LPS. However, after treatment with GOS1-4, levels of inflammatory mediators (NO, IL-1β, IL-6, and TNF-α) were markedly decreased, indicating the following trend of anti-inflammatory activity: GOS4 > GOS3 > GOS2 > GOS1 (Figure 3). GOS4, which had the highest polymerization degree of tested samples, produced the strongest inhibition of NO, IL-1β, TNF-α, and IL-6 secretion. These results indicated that the degree of polymerization affected the anti-inflammatory activity of GOS: the higher the polymerization degree, the stronger the anti-inflammatory activity. Likewise, qPCR confirmed a consistent trend in anti-inflammatory activity for mRNA expression levels of pro-inflammatory factors IL-1β, IL-6, and TNF-α (Figure 4). These pro-inflammatory enzymes’ mRNA expression also suggested that GOS4 had higher anti-inflammatory activity in regulating inflammation in vitro (Figure 5).

**Figure 8:**
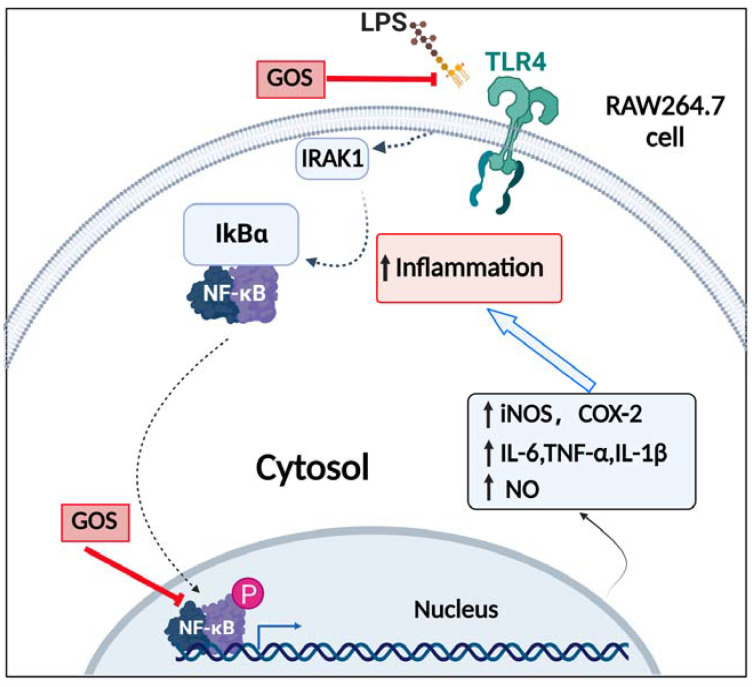
Schematic of the signaling pathway involved in anti-inflammatory actions elicited by GOS in RAW264.7 cells. Solid arrows indicate the pathway examined, while dotted arrows indicate pathway components not examined in the present study. P, phosphorylation.

TLR4 is one of the most important ligands of LPS, and the TLR4 signaling pathway activated by LPS plays an important role in inflammatory signal transduction [37,38]. Phosphorylation of several protein kinases related to the NF-κB signaling pathway, such as IRAK-1 and IKKs, upon binding of LPS to TLR4, leads to the nuclear translocation of NF-κB [39,40]. As is known to all, the NF-κB component exists in the cytoplasm as an inactive form under normal conditions [41,42]. However, upon stimulation by LPS, IκBα is activated by phosphorylation and degraded, which allows free p-p65 to enter the nucleus and mediate downstream signaling target genes of NF-κB activation [43,44]. Hence, suppressing the activation of the TLR4/NF-κB pathway could be an effective treatment for inflammation. In the present research, western blot analysis made clear that the induction of inflammation by LPS activated TLR4, resulting in phosphorylation and degradation of IκBα in cells. In contrast, GOS suppressed LPS-induced TLR4 expression, IκBα phosphorylation, NF-κB phosphorylation, and subsequent nuclear translocation in cells (Figure 6). These in vitro results further demonstrated that GOS could effectively inhibit LPS-induced TLR4/NF-κB activation. A schematic of the potential mechanism of action (Figure 8) indicates that GOS suppressed the protein expression of TLR4, p-IκBα, and p-p65 to inhibit downstream gene expression and thus attenuate inflammation activation induced by LPS in RAW264.7 macrophages. Meanwhile, our study demonstrates that GOS suppressed expression of pro-inflammatory mediators such as IL-1β, IL-6, and TNF-α. Moreover, GOS significantly reduced COX-2 and iNOS mRNA expression, which was accompanied by decreased NO levels. To sum up, this research indicated that GOS4 (with the highest polymerization degree) could exert the strongest anti-inflammatory activity in suppressing the TLR4/NF-κB pathway.

In present years, increasing attention has been paid to the potential nutrition and health benefits of various food ingredients. As a functional oligosaccharide, GOS was one of the important prebiotics, which was beneficial to intestinal microecological balance. This study elucidated the relationship between GOS polymerization degree and anti-inflammatory activity and confirmed the in vitro anti-inflammatory effects of GOS via the TLR4/NF-κB pathway, which provided theoretical guidance for the development of new prebiotics. However, we only investigated the relationship between polymerization degree and anti-inflammatory activity at the cellular level, which is still worth further exploration at the animal level. In addition, further in-depth quantitative structure-activity relationship research is needed with the help of computer-aided tools. Therefore, it is necessary for us to explore these unsolved issues further in the future.

## 5. Conclusions

The present study highlighted that GOS could inhibit the TLR4/NF-κB pathway activation to exert an anti-inflammatory effect. More importantly, the degree of GOS polymerization has an important influence on their activity: the higher the polymerization degree, the stronger the anti-inflammatory activity. Our results showed that GOS downregulated levels of pro-inflammatory cytokines (IL-1β, IL-6 and TNF-α) and enzymes (iNOS and COX-2) to exert anti-inflammatory activity in LPS-induced inflammation. In addition, diverse GOS differentially reduced LPS-induced inflammation in macrophages. In particular, GOS4 had the strongest anti-inflammatory activity among GOS1-4. This further indicated that it is expected to provide a certain data reference for the development of functional foods, and GOS manufacturers could optimize technology to increase the content of GOS of higher polymerization degree (i.e., GOS4) if they want to create products with tailor-made anti-inflammatory activity.

## Figures and Tables

**Table 1:** Sequences of Real-time PCR primers.

Genes	Forward Primer (5′-3′)	Reverse Primer (5′-3′)
iNOS	CAGCGGAGTGACGGCAAACAT	GCAAGACCAGAGGCAGCACATC
IL-1β	ACCTGGGCTGTCCTGATGAGAG	TGTTGATGTGCTGCTGCGAGAT
COX-2	CTGGTGCCTGGTCTGATGATGTATG	TCTCCTATGAGTATGAGTCTGCTGGTT
TNF-α	TGGAACTGGCAGAAGAGGCACT	AGAGGCTGAGACATAGGCACCG
IL-6	GTTCTCTGGGAAATCGTGGA	GGAAATTGGGGTAGGAAGGA
GAPDH	ACTCCACTCACGGCAAATTC	GTCATGAGCCCTTCCACAAT

**Table 2:** NO, IL-1β, TNF-α, and IL-6 levels analysis of culture media obtained from cells treated with LPS and natural GOS.

Groups	NO (μmol/L)	IL-1β (pg/mL)	TNF-α (ng/mL)	IL-6 (ng/mL)
Control	1.18 ± 0.61 ^f^	24.04 ± 0.20 ^f^	1.49 ± 0.12 ^f^	0.24 ± 0.01 ^f^
LPS	14.14 ± 0.19 ^a^	88.09 ± 0.26 ^a^	121.64 ± 1.29 ^a^	71.48 ± 0.12 ^a^
Ber	12.91 ± 0.47 ^b^	65.66 ± 0.18 ^b^	85.83 ± 0.28 ^b^	55.84 ± 0.21 ^b^
Raffinose	10.83 ± 0.12 ^c^	65.17 ± 0.29 ^c^	97.18 ± 0.29 ^c^	67.16 ± 0.23 ^c^
Stachyose	10.30 ± 0.18 ^d^	64.13 ± 0.11 ^d^	93.90 ± 0.24 ^d^	60.90 ± 0.13 ^d^
Verbascose	7.21 ± 0.08 ^e^	55.32 ± 0.29 ^e^	92.50 ± 1.02 ^e^	56.80 ± 0.22 ^e^

Superscript lowercase letters indicate statistically significant differences (*p* < 0.05).

## Data Availability

The data presented in this study are available on request from the corresponding author.
